# An integrated “Engage & Evasion” approach for mononuclear phagocyte system escape and efficient extracellular vesicle therapy

**DOI:** 10.1186/s12951-024-03032-z

**Published:** 2024-12-19

**Authors:** Hongman Liu, Mengting Li, Bing Xiang, Ziying Yang, Shiyu Cao, Wen Gong, Jingjing Li, Wenjing Zhou, Liang Ding, Qingsong Tang, Shengnan Wang, Jin Tang, Zixuan Fan, Ke He, Xuan Jiang, Zhenya Shen, Weiqian Chen, Jie Hui

**Affiliations:** 1 Department of Cardiovascular Surgery of The First Affiliated Hospital & Institute for Cardiovascular Science, Suzhou Medical College, Soochow University, Suzhou, China; 2 Department of Cardiology, The First Affiliated Hospital of Soochow University, Suzhou, China; 3 Department of Cardiovascular Medicine, The Affiliated Taian City Central Hospital of Qingdao University, Taian, China; 4 Shenzhen Key Laboratory for Systems Medicine in Inflammatory Diseases, School of Medicine, Sun Yat-Sen University, Shenzhen, China; 5 School of Life Sciences, Faculty of Medicine, Tianjin University, Tianjin, China; 6 Hangzhou Institute of Medicine (HIM), Chinese Academy of Sciences, Hangzhou, China

**Keywords:** Ischemic disease, Extracellular vesicle, Mononuclear phagocyte system, CD47, Phagocytosis

## Abstract

Ischemic diseases are major contributors to global morbidity and mortality, posing a substantial threat to human health. Extracellular vesicles (EVs) play an essential role in enhancing neovascularization in ischemic tissues, thereby facilitating tissue repair and regeneration. However, the utilization of EVs is hindered by their rapid uptake and clearance by the mononuclear phagocyte system (MPS), which markedly impedes their therapeutic efficacy and organ-specific accumulation. Notably, CD47, upon binding to signal regulatory protein alpha, initiates a “don’t eat me” signal, enabling immune evasion from the MPS. Our research has demonstrated that phagocytes predominantly engulf CD47^low^ dendritic DC2.4 cell-derived EVs (DV), while engineered CD47^high^ EVs (MV^47^) experience minimal ingestion. Leveraging these findings, we have developed a dual-faceted “Engage & Evasion” strategy. Initially, DVs were employed to saturate the MPS, serving as the “engage” component. Subsequently, MV^47^, fortified with CD47, was introduced for “evasion” purposes. This approach effectively minimized entrapment by the liver and spleen, boosted serum concentration, and enhanced final accumulation in non-MPS organs. In summary, our “Engage & Evasion” therapeutic strategy offers a promising avenue to enhance EV therapeutic potential against ischemic challenges through improved systemic distribution.

**Supplementary Information:**

The online version contains supplementary material available at 10.1186/s12951-024-03032-z.

## Introduction

Ischemic conditions, which significantly contribute to global health issues and mortality, present a major challenge to health systems worldwide. In response to this, extracellular vesicles (EVs), tiny membrane-bound vesicles released by living cells, have been recognized as a promising therapeutic approach due to their proficiency in intercellular communication [1, 2]. Accumulating evidence suggests that EVs, carrying distinct proteins and nucleic acids from their source cells, are crucial in formation of new blood vessels in ischemic regions, thereby aiding the process of tissue healing and regeneration [3].

Nevertheless, the therapeutic application of EVs faces challenges, particularly their rapid clearance from the bloodstream [4, 5]. This clearance, primarily orchestrated by the mononuclear phagocyte system (MPS) in organs like the liver and spleen, involves the swift removal of foreign entities, posing a significant barrier to effective therapeutic delivery [6]. To overcome this hurdle, strategies have been developed to utilize the protective function of CD47, a critical membrane protein that signals macrophages through the signal regulatory protein alpha (SIRPα) to avoid phagocytosis, effectively sending a “don’t eat me” signal. Surface modification of cells or nanoparticles with CD47 has demonstrated efficacy in extending the circulation time of drugs [7]. However, despite biomimetic approaches that employ CD47 overexpression to create a “stealth” coating on cell membranes and evade MPS detection, a fraction of these nanoparticles is still rapidly cleared due to extensive interactions within the physiological environment [8]. Consequently, there is an urgent need for more robust interventions to counteract MPS clearance.

**Scheme 1:**
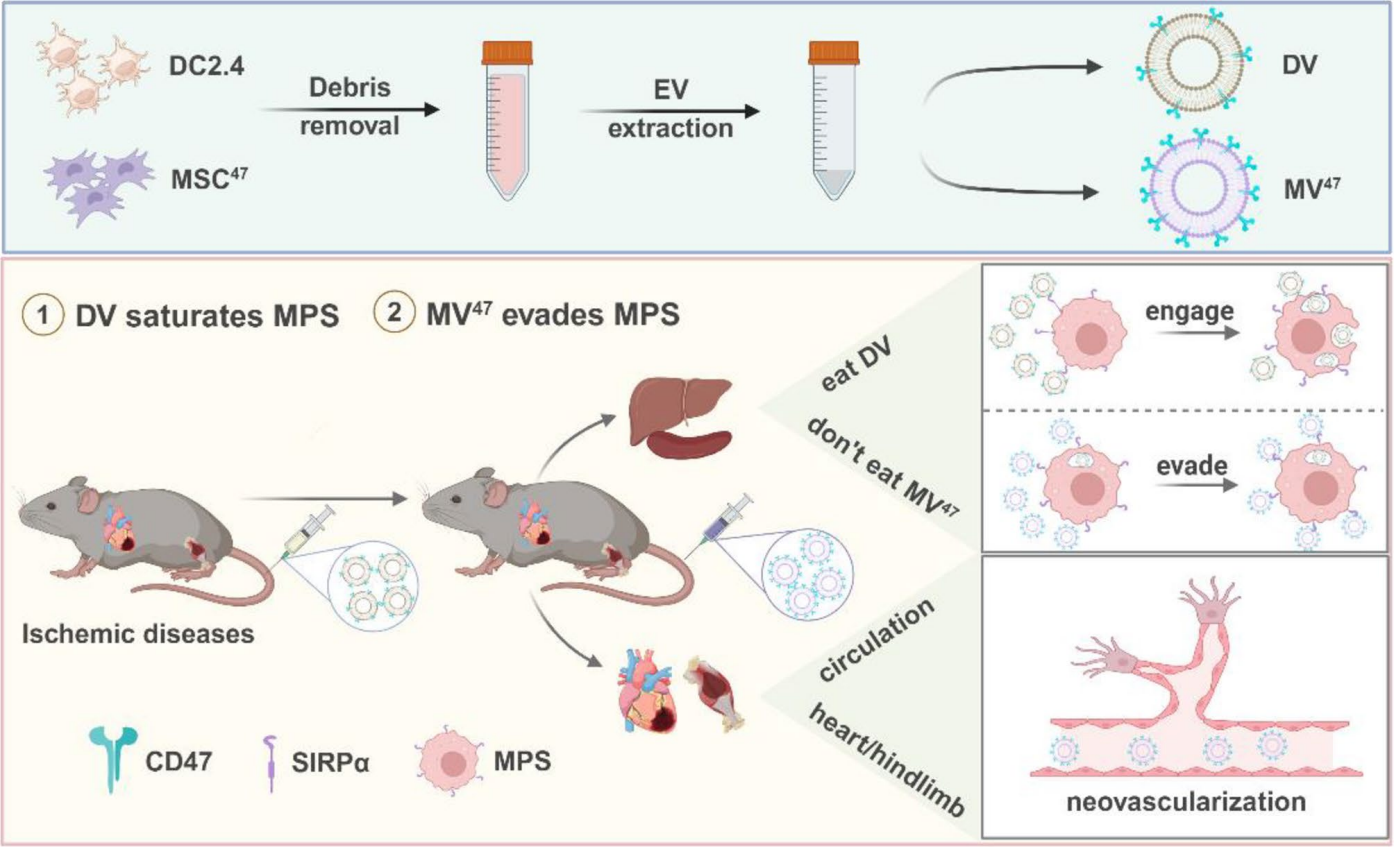
Overview of “Engage & Evasion” approach benefiting ischemic diseases. Created with BioRender.com

In this study, we devised a novel administration strategy aimed at minimizing EV entrapment by the liver and spleen, particularly for ischemic disease treatment. Notably, EVs from DC2.4 cells (DV) demonstrated a higher affinity for the MPS than those from other sources. Our approach began with the DV prime to saturate the MPS, acting as an “engage” signal. Subsequently, the strategy shifted to “evasion” tactics. This involved utilizing CD47^high^ EVs (MV^47^) to hinder the phagocytosis of therapeutic EVs. Our dual-phase “engage and evade” strategy aims to enhance the circulation longevity and therapeutic efficacy of EVs, showing superior outcomes compared to those achieved with conventional single-injection techniques (Scheme 1).

## Results and discussion

### Characterization and identification of EVs with distinct CD47 expression profiles

**Fig. 1:**
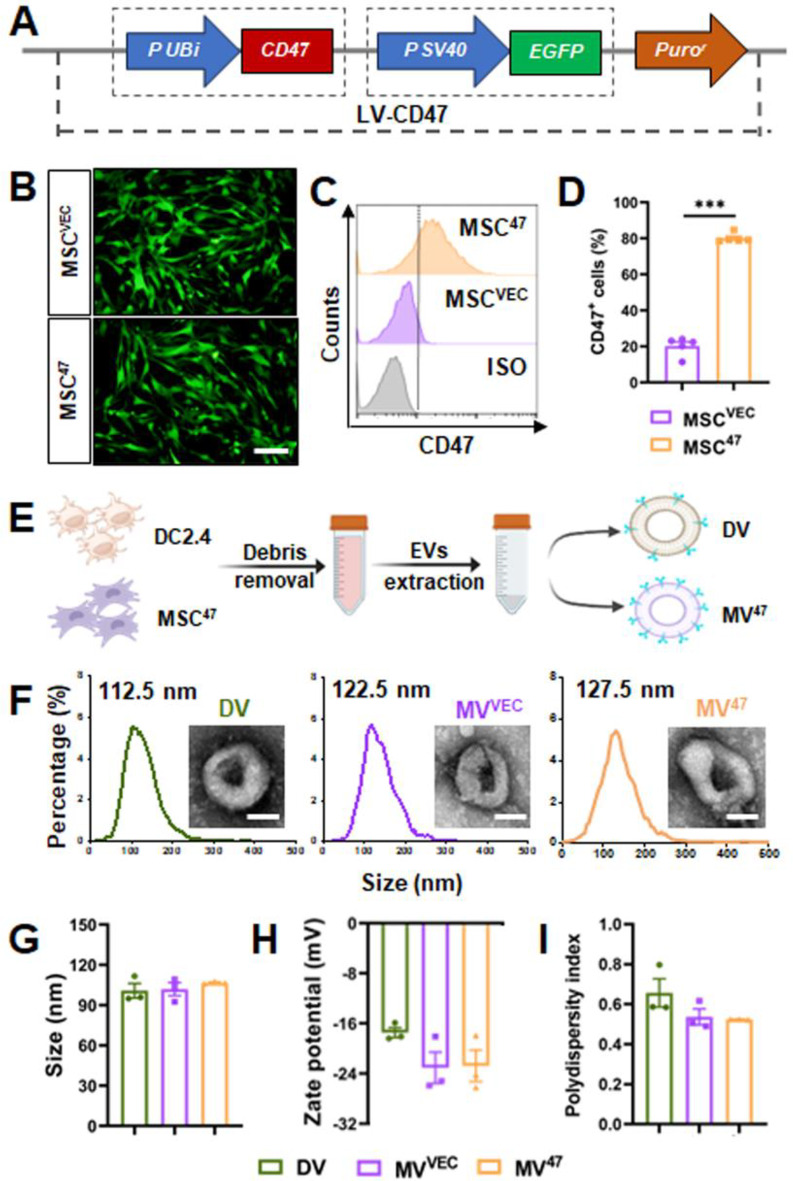
Characterization and identification of designed EVs. (**A**) Schematic of CD47-overexpressing lentiviral construct. (**B**) Cytosolic EGFP fluorescence observed under inverted fluorescence microscopy. Scale bar = 100 μm. (**C**&**D**) Flow cytometric histograms and quantification for CD47 expression (*n* = 5). (**E**) Schematic diagram of EV extraction. (**F**) EV morphology and size distribution were examined using transmission electron microscopy (TEM) and nanoparticle tracking analysis (NTA), respectively. Scale bar = 50 nm. (**G**-**I**) Size distribution, zeta potential, and polydispersity index (PDI) were determined by dynamic light scattering. Statistical evaluations were conducted using a two-tailed unpaired Student’s t-test. Results were presented as mean ± SEM (*** *P* < 0.001)

CD47, a transmembrane protein ubiquitously expressed in various cell types, interacts with SIRPα on macrophages, a crucial interaction for inhibiting macrophage-mediated phagocytosis [9]. Given this mechanism, we hypothesized that membranous CD47 might activate the CD47/SIRPα axis to evade macrophage engagement. To investigate this, we utilized lentiviral transduction to engineer mesenchymal stem cells (MSCs) to overexpress membranous CD47 and cytosolic EGFP, followed by selection with puromycin (Fig. 1A). The presence of cytosolic EGFP confirmed successful transduction (Fig. 1B), and flow cytometry validated the overexpression of CD47 (Fig. 1C&D), indicating the successful generation of a CD47-overexpressing MSC line.

To isolate EVs with distinct CD47 expression profiles, EVs were harvested from culture media of DC2.4 and MSC^47^ cells (Fig. 1E). Initially, all EVs displayed a predominant saucer-shaped structure with a lipid bilayer, exhibiting a consistent size distribution mainly ranging from 100 to 200 nm, with peak sizes observed at 112.5, 122.5, and 127.5 nm (Fig. 1F&G, Figure S1). Importantly, dynamic light scattering analysis further revealed that all EVs maintained an average zeta potential within the range of -15 to -30 mV (Fig. 1H) and possessed a polydispersity index (PDI) around 0.5 (Fig. 1I). Collectively, these findings demonstrated that our engineered EVs maintain uniform morphological, size, and surface characteristics.

### Characterization of biological marker expression patterns using interferometric imaging

**Fig. 2:**
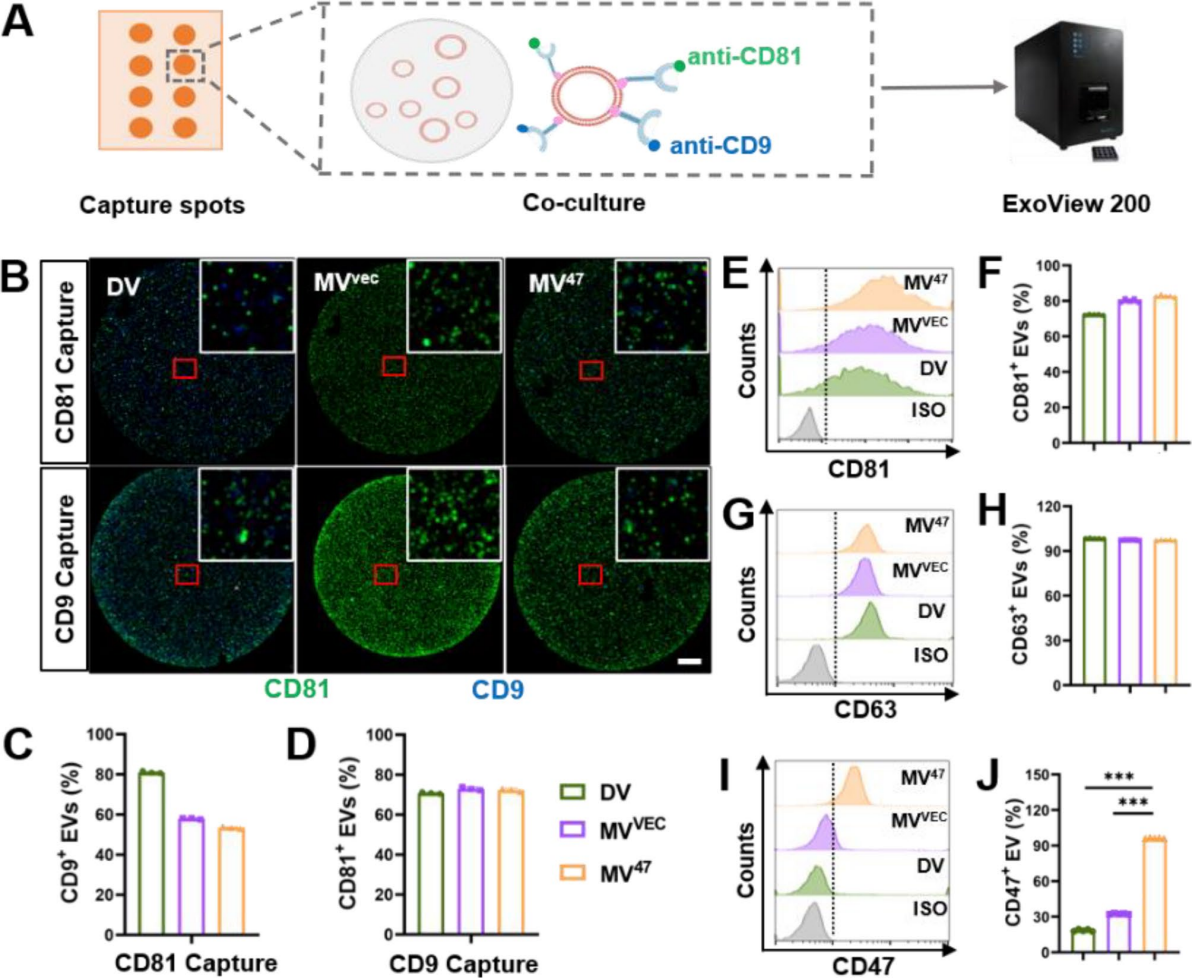
Expression of CD47 and common markers on EV Surfaces. (**A**) Schematic diagram depicting ExoView 200-based EV biological characterization. (**B**) Representative images demonstrating co-localization of CD81 and CD9 on individual EVs. An enlarged image is displayed in the top right corner to highlight details. Scale bar = 20 μm. (**C**&**D**) Quantification of CD9^+^ and CD81^+^ EVs from CD81 or CD9 capture spots. (**E**-**J**) Aldehyde/sulfate latex beads-based flow cytometric histograms detail CD81, CD63, and CD47 expression on individual EVs (*n* = 5). Statistical evaluations were conducted using one-way ANOVA. Results were presented as mean ± SEM (*** *P* < 0.001)

To investigate the biological characteristics across different EVs, we utilized the ExoView 200, an advanced interferometric imaging platform designed for capturing, isolating, and imaging individual nanoparticles within complex matrices. This system employs antibody microarrays for high-affinity target capture [10]. Our approach involved coating silicon chips with antibodies targeting murine CD81 and CD9 (Fig. 2A), generating images that demonstrated clear co-localization of these markers (Fig. 2B). The presence of CD9 and CD81 was uniformly confirmed in both CD81 and CD9 capture spots (Fig. 2C&D), affirming their stable expression across all EV populations. Furthermore, Flow cytometry confirmed a consistent pattern of CD81 and CD63 expression across the examined cohorts (Fig. 2E-H). Notably, the highest CD47^+^ ratio in the MV^47^ group and the lowest in the DV group were also observed (Fig. 2I&J). Compared to alternatives like PEGylation [11] for extending circulation time, our CD47-based genetic engineering offers a more favorable solution. It provides benefits such as low toxicity, high stability, significant scalability, and cost-effectiveness. Collectively, these results highlighted differential CD47 expression while verifying the consistent presence of CD81 and CD9 across all EV types, thus substantiating the distinct profiles of these EV populations.

### Internalization efficiency of different EVs

**Fig. 3:**
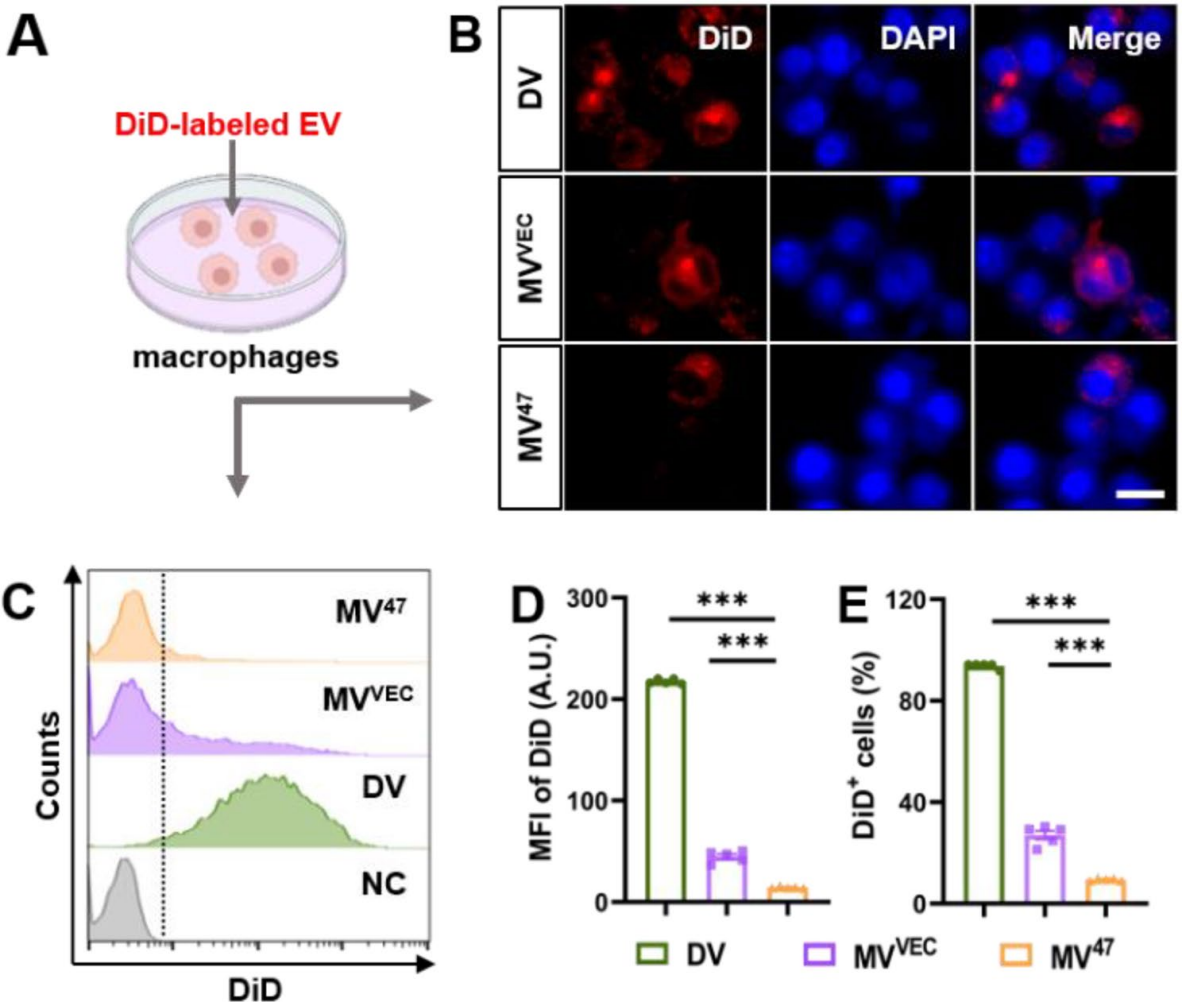
Differential Uptake of DV and MV^47^ by Macrophages. (**A**) The schematic diagram for in vitro uptake of EVs. DiD-labeled EVs were exposed to RAW264.7 macrophages for 4 h. (**B**) Representative immunofluorescent images showing EV uptake. Scale bar = 10 μm. (**C**-**E**) Flow cytometric histograms and quantification for DiD signals (*n* = 5). Statistical evaluations were conducted using one-way ANOVA. Results were presented as mean ± SEM (*** *P* < 0.001)

Given the role of the CD47-SIRPα axis in immune evasion [12], we explored the myeloid internalization of DV and MV^47^. Both types of EVs were labeled with DiD and co-cultured with RAW264.7 macrophages (Fig. 3A). Immunofluorescence analysis revealed that DVs were predominantly internalized by target cells, whereas MV^47^ exhibited significantly lower uptake (Fig. 3B, Figure S2). Flow cytometry analysis further validated that DVs were internalized more effectively than MV^47^ (Fig. 3C-E), suggesting distinct mechanisms of “Engage” and “Evasion” in myeloid cells.

### “Engage & Evasion” strategy inhibited MPS entrapment and promotes serum biodistribution

**Fig. 4:**
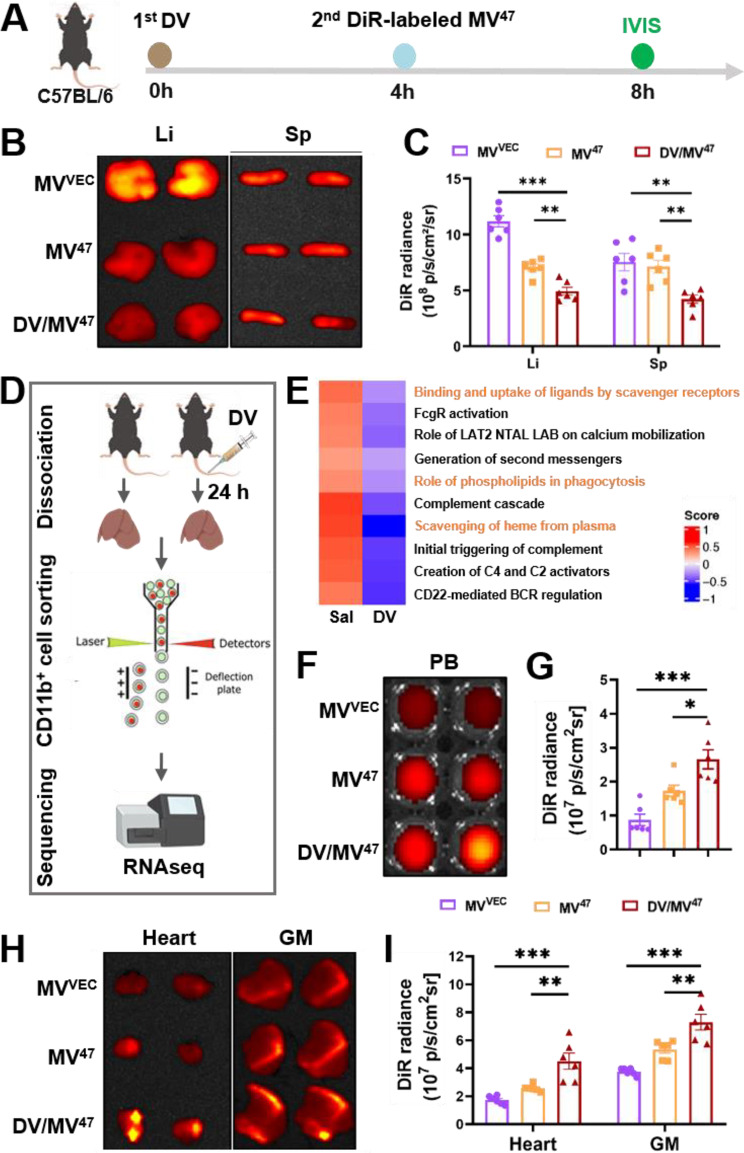
In vivo biodistribution of therapeutic MV^47^. (**A**) Schematic illustration of in vivo testing: C57BL/6 mice received tail vein injections of DiR-labelled MV^VEC^ and MV^47^, with or without DV pre-blocking, for subsequent in vivo tracking. (**B**&**C**) Representative DiR optical images alongside quantification of biodistribution in the liver and spleen (*n* = 6). (**D**) Schematic detailing the sample preparation process for RNAseq. (**E**) Gene set variation analysis (GSVA) was conducted on macrophage activity-related Reactome pathways, highlighting phagocytosis-related pathways in orange. (**F**-**I**) Representative DiR optical images and accompanying quantification for biodistribution in the peripheral blood (**F**&**G**), heart, and gastrocnemius muscle (**H**&**I**) (*n* = 6). Li, liver; Sp, spleen; PB, peripheral blood; GM, gastrocnemius muscle. Statistical evaluations were conducted using one-way ANOVA. Results were presented as mean ± SEM (* *P* < 0.05; ** *P* < 0.01; *** *P* < 0.001)

In agreement with previous research, the therapeutic efficacy of EVs has been notably restricted due to rapid clearance by the mononuclear phagocyte system (MPS), predominantly in the MPS-rich liver and spleen [13]. To counteract this limitation, we set up an innovative “Engage & Evasion” strategy aimed at reducing EV phagocytosis by MPS-enriched organs. We first administered DV to initiate the “eat me” signal as the “engage” component. Subsequently, we injected DiR-labeled MV^47^, while ensuring that its physiochemical characteristics remained unaltered (Figure S3), as the “don’t eat me” signal to initiate the “evasion” component. Organ collection was conducted four hours after sequential treatment (Fig. 4A). As anticipated, IVIS ex vivo imaging revealed significantly weaker DiR signals in the liver and spleen following DV pre-blockade (Fig. 4B&C), indicating effective MPS suppression. Subsequent RNA sequencing of DV-primed Kupffer cells provided a comprehensive, genome-wide perspective (Fig. 4D, Figure S4A), revealing a marked reduction in their phagocytic activity (Fig. 4E, Figure S4B). Moreover, DV-primed Kupffer cells exhibited significantly reduced activity in macrophage-related pathways (Reactome database), including the FcgR-dependent ADCC (antibody-dependent cellular cytotoxicity), complement activation, and red blood cell clearance. Key genes associated with myeloid phagocytosis and activation, such as UDP glucuronosyltransferase 1 family, polypeptide A1 (*Ugt1a1*), and Exosome Component 6 (*Exosc6*) were predominantly downregulated (Figure S4C). Notably, *Ugt1a1* facilitates the metabolism and excretion of lipophilic molecules [14], while *Exosc6* is a non-catalytic subunit of the exosome complex, playing an important role in RNA metabolism and catabolism [7]. Additionally, we also validated several candidate genes in DV-primed RAW264.7 cells and observed consistent expression of *Pde2a*, *Pkd1*, and *Creb5* with RNAseq results from DV-primed Kupffer cells (Figure S4D). Consequently, peak serum concentrations of DiR-labeled MV^47^ were observed in the “Engage & Evasion” group (Fig. 4F&G), with notable accumulation in organs prone to ischemia, such as the heart and gastrocnemius muscle (Fig. 4H&I), as well as in non-ischemic organs, such as the lung (Figure S5). These findings suggested that the “Engage & Evasion” strategy not only diminishes MPS-mediated phagocytosis but also enhances the delivery of therapeutic EVs to specific organs, underscoring its potential for improving the efficacy of EV-based therapies.

### “Engage & Evasion” strategy accelerates cardiac function recovery post-MI

**Fig. 5:**
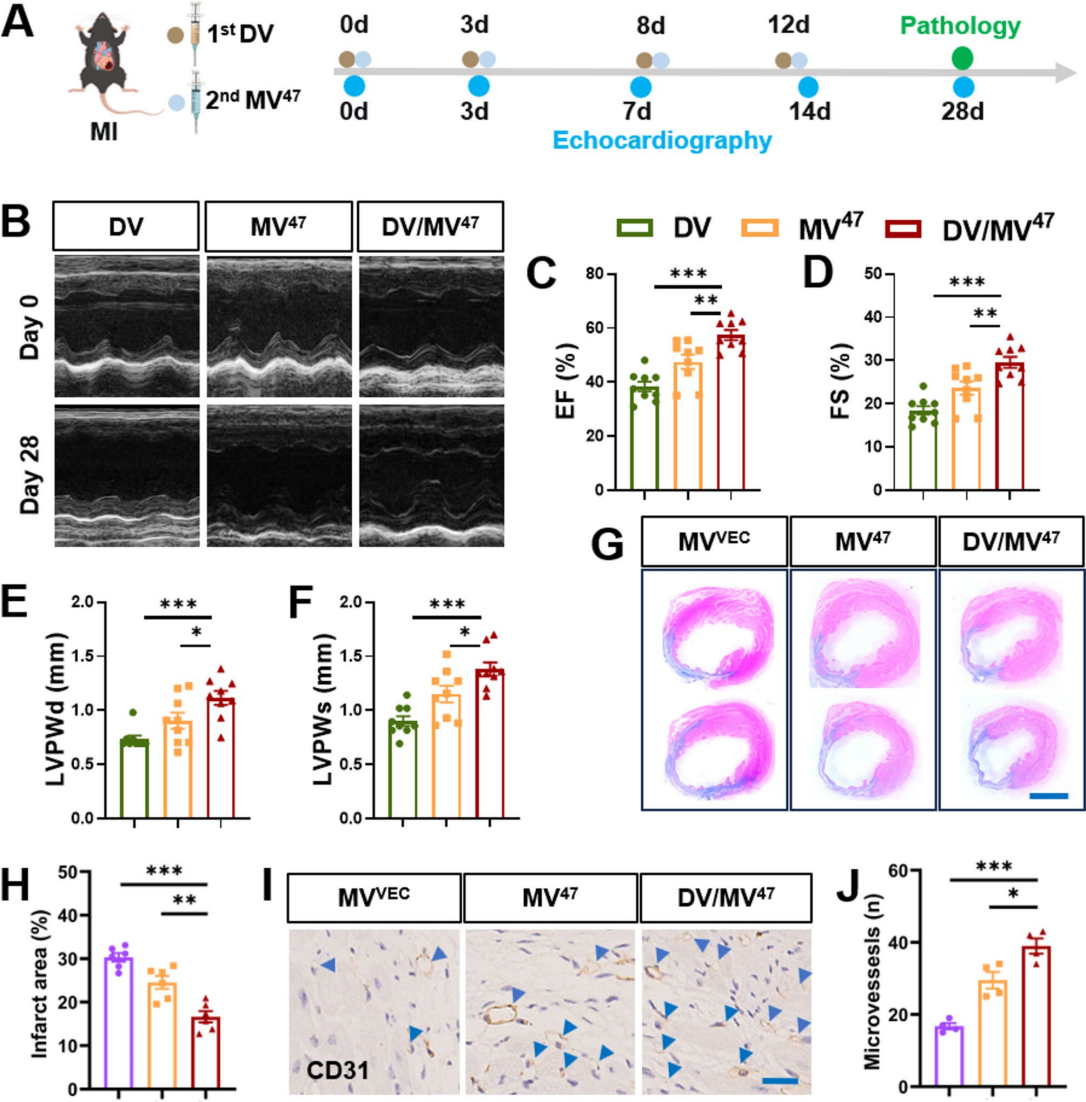
Mitigation of post-MI cardiac remodeling by “Engage & Evasion” approach. (**A**) Schematic experimental design. (**B**) M-mode echocardiographic images captured on Days 28 post-MI. (**C**-**F**) Bar charts for cardiac function and structure, including metrics such as left ventricular ejection fraction (EF) and fractional shortening (FS), as well as systolic (LVPWs) and diastolic (LVPWd) posterior wall thicknesses (*n* = 9). (**G**&**H**) Masson’s trichrome staining and quantification (*n* = 6). Scale bar = 2 mm. (**I**&**J**) Representative IHC staining for CD31 and corresponding quantification (*n* = 4). Blue arrowheads highlight CD31^+^ capillaries. Scale bar = 25 μm. Statistical evaluations were conducted using either two-way ANOVA or one-way ANOVA. Results were presented as mean ± SEM (* *P* < 0.05; ** *P* < 0.01; *** *P* < 0.001; *ns*: not significant)

MSCs have been acknowledged for their ability to mediate cardioprotective paracrine effect against myocardial ischemia/reperfusion injury through secretion of EVs [15], highlighting a new perspective in the advancement of tissue repair biologics. In our study, we assessed the “Engage & Evasion” strategy’s efficacy in facilitating recovery from myocardial infarction (MI)-induced cardiac dysfunction. After permanently ligating the left anterior descending artery, mice received tail vein injections of designated EVs, with subsequent assessments of cardiac function and pathology at specified time points (Fig. 5A). Specifically, the DV-primed group exhibited significant increases in left ventricular ejection fraction and fractional shortening (Fig. 5B-D), indicative of ameliorated ventricular dysfunction. Increases in both systolic (LVPWs) and diastolic (LVPWd) posterior wall thicknesses further confirmed these findings (Fig. 5E&F). Histological examinations, supported by Masson’s trichrome and CD31 immunohistochemical staining, displayed minimal scar tissue formation (Fig. 5G&H) and increased CD31^+^ capillary counts (Fig. 5I&J). Compared other EV-based therapies, like cargo-loading [6] or drug pretreatment [16], cardioprotective efficacy of our “Engage and Evasion” therapy remains uncompromised due to optimal utilization of EVs. Collectively, these results demonstrated that our “Engage and Evasion” therapy not only mitigates cardiac dysfunction but also actively stimulates neovascularization, thereby supporting cardiac repair post-MI.

### “Engage & Evasion” strategy promotes recovery of ischemic hindlimbs

**Fig. 6:**
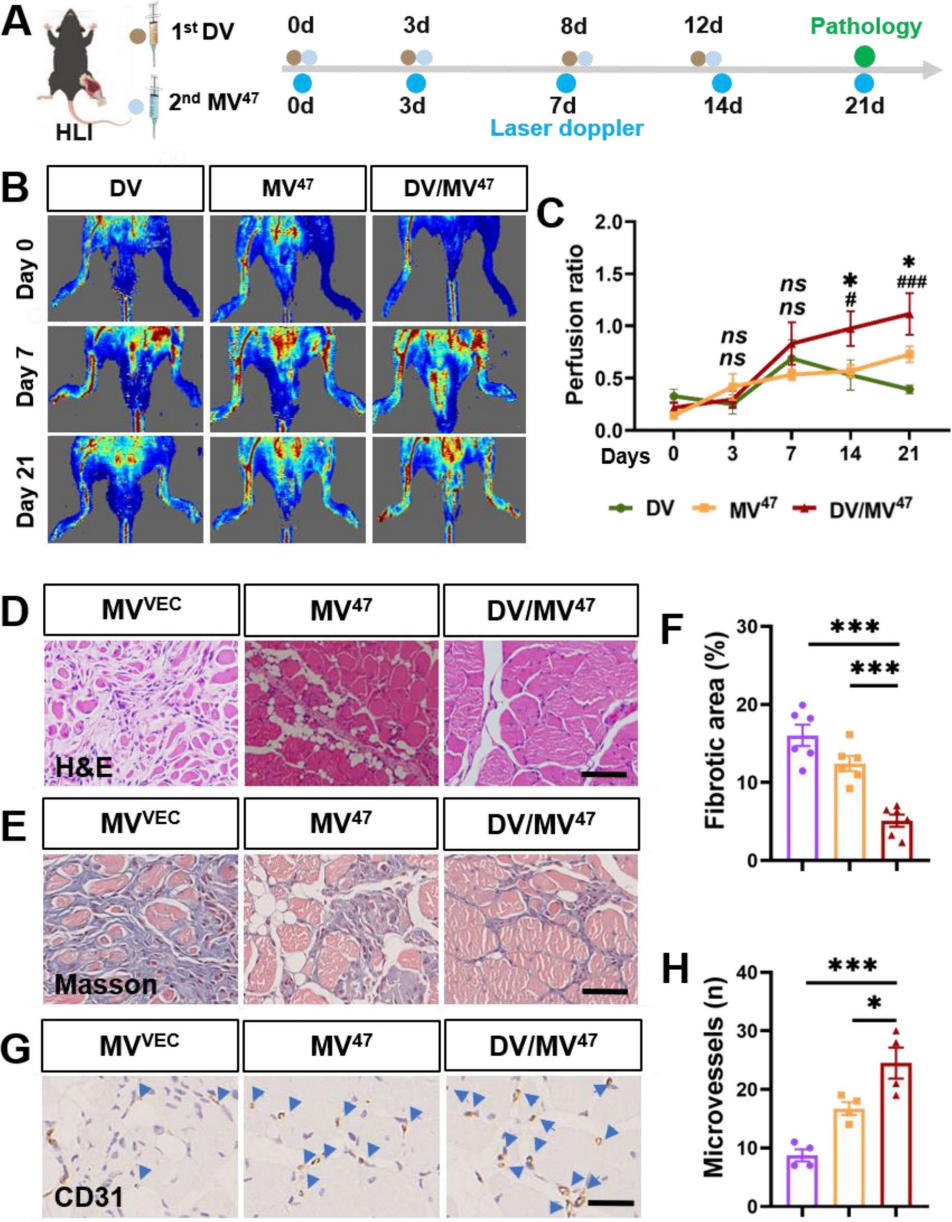
Accelerated recovery from post-hindlimb ischemia using “Engage & Evasion” approach. (**A**) Schematic experimental protocol. (**B**&**C**) Laser Doppler imaging with perfusion ratio quantification (*n* = 6). * for comparison between DV/MV^47^ and MV^47^ group; # for comparison between DV/MV^47^ and DV group. (**D**) Representative H&E images. Scale bar = 50 μm. (**E**&**F**) Masson’s trichrome staining with quantification depicting interstitial fibrosis (*n* = 6). Scale bar = 50 μm. (**G**&**H**) IHC staining for CD31 with corresponding quantification. Blue arrowheads indicate CD31^+^ capillaries (*n* = 4). Scale bar = 25 μm. Statistical evaluations were conducted using either two-way ANOVA or one-way ANOVA. Results were presented as mean ± SEM. (*# *P* < 0.05; ***### *P* < 0.001; *ns*: not significant)

To further assess the efficacy of “Engage & Evasion” therapy in enhancing blood perfusion and promoting angiogenesis, we employed a murine hindlimb ischemia model (Fig. 6A). Treatment with MV^47^ significantly facilitated recovery in hindlimb blood perfusion, which was notably augmented by DV priming. This enhancement was quantitatively evidenced by an increase in blood flow on Day 21 post-ischemia, as measured using laser Doppler perfusion imaging (Fig. 6B&C). Additionally, histological examination of gastrocnemius muscles on Day 21 post-ischemia revealed a pronounced reduction in immune cell infiltration (Fig. 6D) and interstitial fibrosis (Fig. 6E&F) within the “Engage & Evasion” group. Finally, increased neovascularization was also confirmed by CD31 immunohistochemical staining (Fig. 6G&H), indicating maximal angiogenic potential by “Engage & Evasion” therapy. Collectively, these findings suggested the extensive muscle protection and enhanced therapeutic outcomes facilitated by the strategy.

### “Engage & Evasion” strategy boosts endothelial function by limiting myeloid uptake

**Fig. 7:**
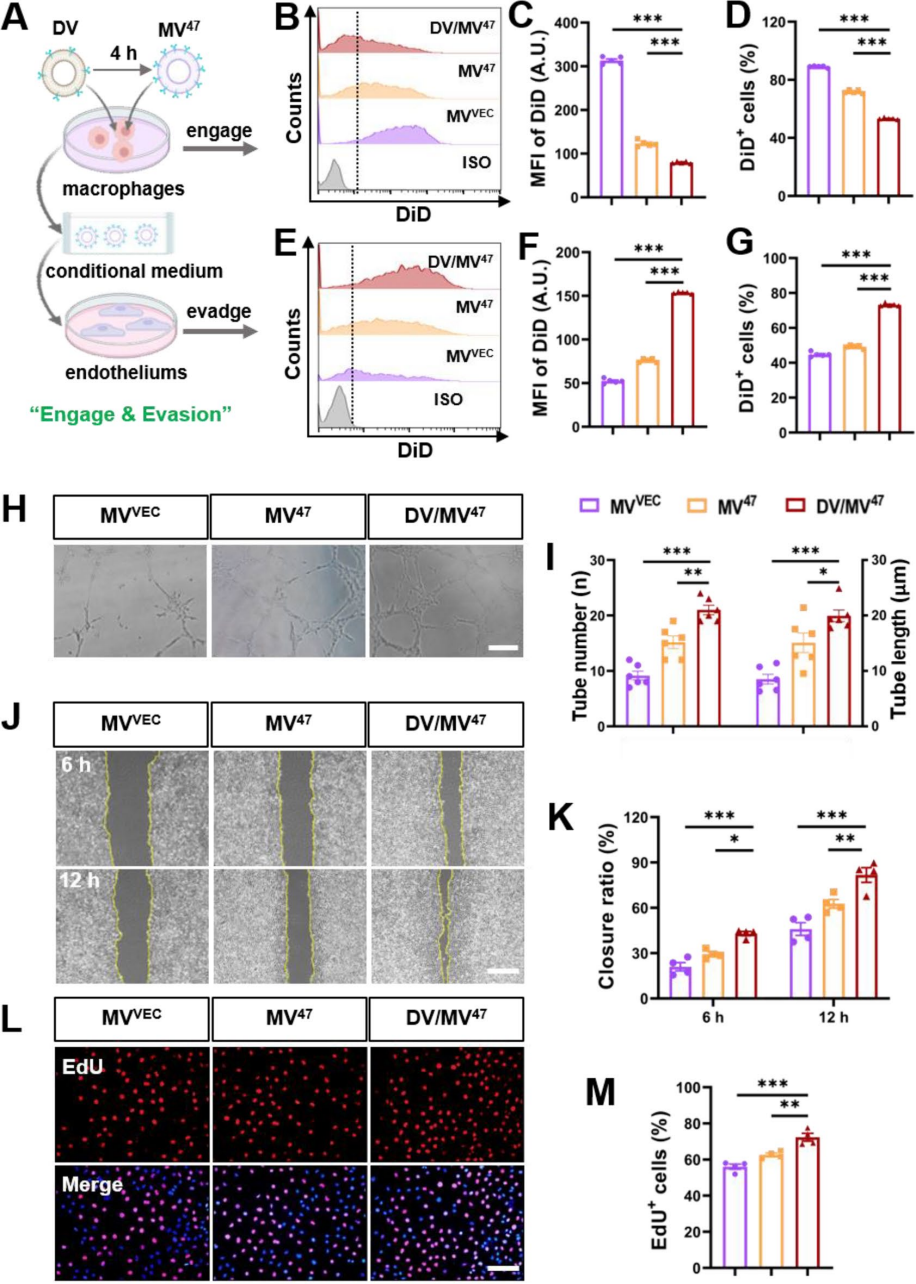
“Engage & Evasion” strategy boosts endothelial proangiogenic efficacy by MPS evasion. (**A**) Schematic diagram illustrating “Engage & Evasion” strategy in vitro. Initially, RAW264.7 macrophages were exposed to DV for 4 h to mimic MPS phagocytosis. Subsequently, therapeutic MV^47^ was administered for another 4 h. The resultant conditional medium was then applied to HUVEC endothelial cells. (**B**-**D**) Flow cytometric histograms alongside quantification for DiD signals in RAW264.7 (*n* = 5). (**E**-**G**) Flow cytometric histograms alongside quantification for DiD signals in HUVECs (*n* = 5). (**H**&**I**) Matrigel-based tube formation assay and quantification for tube length and number. Scale bar = 200 μm. (**J**&**K**) Representative images from scratch wound healing assay, alongside quantification for closure ratio (*n* = 4). Scale bar = 100 μm. (**L**&**M**) Representative EdU incorporation images and percentage of EdU^+^ cells. Scale bar = 100 μm. Statistical evaluations were conducted using one-way ANOVA. Results were presented as mean ± SEM (* *P* < 0.05; ** *P* < 0.01; *** *P* < 0.001)

To explore the pro-angiogenetic potential of our “Engage & Evasion” strategy, We developed an in vitro cellular model. Initially, DV was administered to RAW264.7 macrophages; after four hours, therapeutic MV^47^ was introduced (Fig. 7A). Another four hours post-treatment, the macrophages were collected, and analysis via flow cytometry showed a notable decrease in the uptake of DiD-labeled MV^47^ (Fig. 7B-D), indicative of suppressed MPS phagocytosis. Subsequently, endothelial HUVECs were treated with the conditioned medium from these macrophages, now enriched with DiD-labeled MV^47^, which significantly enhanced MV^47^ uptake (Fig. 7E-G). Functionally, the tube formation assay revealed that, although capillary-like structures appeared spontaneously in all groups, the “Engage & Evasion” group displayed a significantly greater number and length of tubes (Fig. 7H&I), denoting superior endothelial function. Additional mechanistic validation provided by the scratch wound healing (Fig. 7J&K) and EdU assays (Fig. 7L&M) demonstrated notable improvements in wound closure and cell proliferation rates, respectively, highlighting enhanced cellular migration and proliferation. Mechanistically, it has been reported that MSC-derived EVs enhance angiogenesis most likely via overexpression and activation of endothelial VEGF receptors [7, 17]. Nonetheless, the underlying mechanism precisely driving neovascularization by our therapeutic MV^47^ requires further investigation. In conclusion, the “Engage & Evasion” strategy effectively enhances the retention and proangiogenic capacity of therapeutic MV^47^ in HUVECs by mitigating MPS-induced phagocytosis.

### Biological safety evaluation of “Engage & Evasion” strategy

**Fig. 8:**
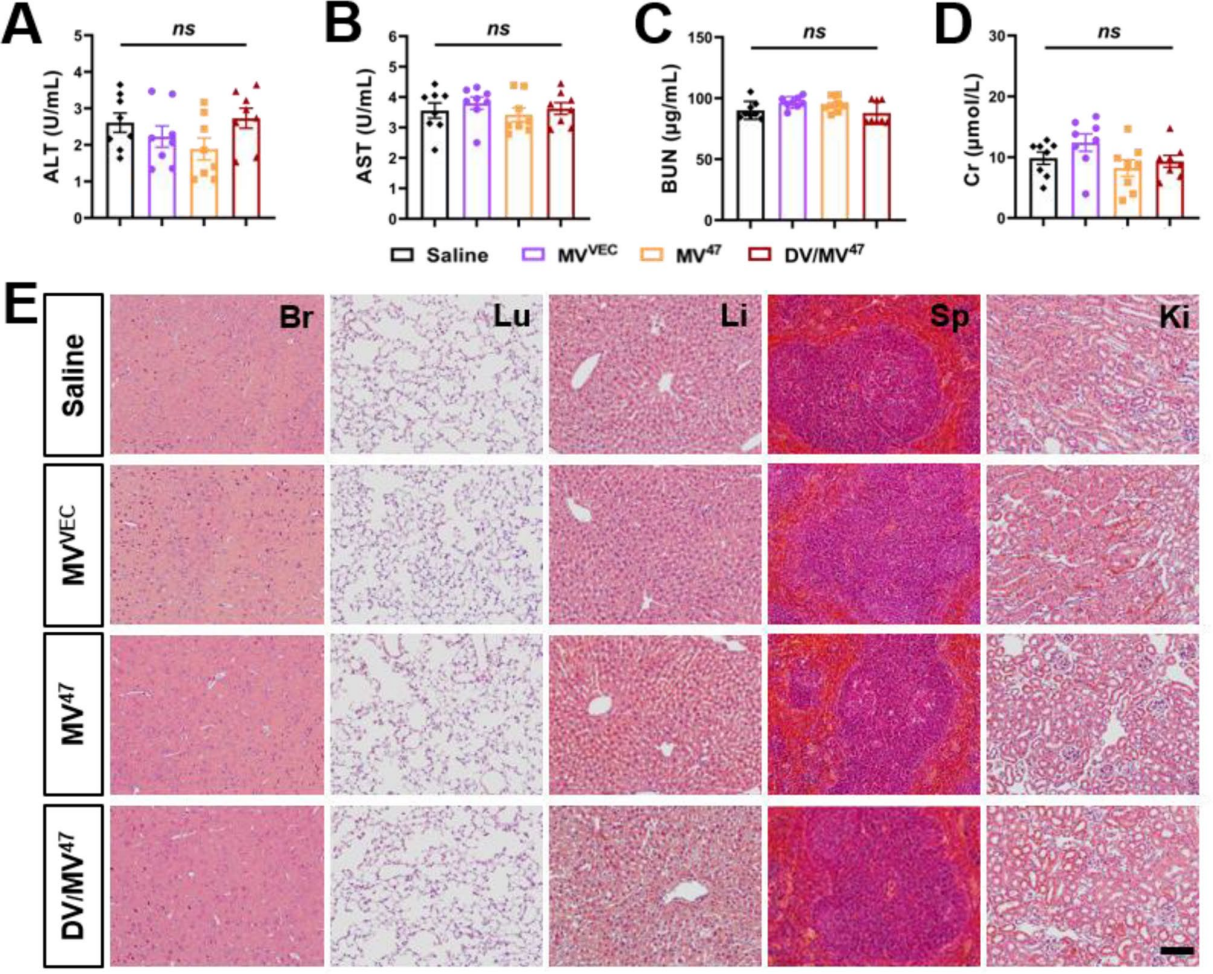
In vivo safety assessment. (**A**-**D**) Serum concentrations of alanine aminotransferase (ALT), aspartate aminotransferase (AST), blood urea nitrogen (BUN), and creatinine (Cr) for hepatorenal toxicity evaluation (*n* = 8). (**E**) H&E stained images of various organs including the brain (Br), lung (Lu), liver (Li), spleen (Sp), and kidney (Ki). Scale bar = 100 μm. Statistical evaluations were conducted using one-way ANOVA. Results were presented as mean ± SEM (*ns*: not significant)

Following intermittent administration over 14 days, all EVs underwent a rigorous safety evaluation [18]. To ascertain the safety profile, we monitored serum concentrations of alanine aminotransferase (ALT), aspartate aminotransferase (AST), blood urea nitrogen (BUN), and creatinine (Cr), which revealed no significant alterations across the evaluated groups (Fig. 8A-D), indicating an absence of hepatorenal toxicity. Additionally, we performed histological examinations on critical organs, including the brain, lung, liver, spleen, and kidney. Crucially, H&E staining verified that our “Engage & Evasion” strategy did not compromise tissue structure, cellular morphology, or immune cell infiltration (Fig. 8E). Collectively, these findings suggested that the “Engage & Evasion” strategy is highly biocompatible and non-toxic, highlighting its potential as a safe therapeutic option in clinical applications.

## Conclusion

In our current study, we have developed a dual “Engage & Evasion” strategy designed to mitigate the phagocytic activity of MPS. This approach began with the administration of DV, followed by the exploitation of therapeutic CD47-enriched MV^47^, renowned for its immune evasion properties. This approach effectively diminished phagocytosis in the liver and spleen, thereby facilitating a greater accumulation of therapeutic EVs in non-MPS tissues. Subsequently, our results demonstrated that the “Engage & Evasion” strategy significantly promotes neovascularization in ischemic tissues, primarily through improving endothelial function. These findings establish a robust basis for advancing therapeutic interventions for ischemic conditions. Nonetheless, it is imperative to acknowledge that certain limitations are still inherent, which require careful consideration in future investigations. Firstly, the endocytic process and molecular biological changes in other organs warrant attention as well. Furthermore, optimizing the cargo loading of appropriate drugs presents an opportunity to enhance therapeutic efficacy.

## Materials and methods

### Cell culture

Murine dendritic DC2.4 cell (Otwo Biotech) and myeloid RAW264.7 cells (kindly provided by Dr. Xuan Jiang from Sun Yat-Sen University, P15-18) were cultured in RPMI 1640 (PrIcella) enriched with 10% fetal bovine serum (ExCell Bio) and 50 µg/mL penicillin/streptomycin. C57BL/6 bone marrow-derived MSCs (Cyagen Biosciences, P8-10) and HUVECs (kindly provided by Dr. Miao Xiao from Soochow University, P8-10) were cultured in commercial MSC Growth Medium (MUXMX-90011, Cyagen Biosciences) and Endothelial Cell Medium (1001, ScienCell), respectively. All cell types were trypsinized using 0.25% Trypsin-EDTA (C100C1, New Cell & Molecular Biotech) and cryopreserved in Serum-Free Cell Freezing Medium (6032011, Dakewe).

### Lentivirus-based cell transduction

Murine CD47-overexpressing lentivirus (GV367, GeneChem) was termed LV-CD47. This lentiviral construct comprises the following vector components: pUbi-CD47-pSV40-EGFP-IRES-puromycin. MSCs infected with LV-CD47 were maintained in the presence of puromycin (5 µg/mL, Solarbio) and designated as MSC^47^. MSCs infected with empty vectors served as controls (MSC^VEC^). Transduction efficiency was evaluated through a fluorescent microscope 72 h post-infection. Overexpression of CD47 was further confirmed by flow cytometry analysis with APC-anti-CD47 (127513, BioLegend) and isotype controls (MultiSciences).

### Isolation and characterization of extracellular vesicles

DC2.4 or transfected MSCs were cultured in a medium supplemented with EV-free FBS. To isolate EVs [19, 20], the cell culture medium was first centrifuged at 2,000 g for 30 min and the supernatant was passed through a 0.22 μm filter to remove dead cells and large debris. Subsequently, this filtered supernatant underwent ultra-centrifugation at 120,000 g for 120 min. Resultant EV pellets were diluted with cold PBS (CW0040S, CoWin) and quantified with Bradford Protein Assay Kit (PA001, Novoprotein).

Particle morphology was assessed using Transmission Electron Microscopy by VivaCell Shanghai Biosciences Co., Ltd (JEM-1200EX, JEOL) or by Wuhan MISP Bio-technology Co., Ltd (JEM1400, JEOL) [21]. Concurrently, particle size distribution was quantified using Nanoparticle Tracking Analysis (Zetaview-PMX120-Z) by Wuhan MISP Bio-technology Co., Ltd. Additionally, measurements of particle diameter and zeta potential were conducted using Dynamic Light Scattering on Zetasizer Nano ZS (Malvern, ZEN3600) [22].

### NanoView microarray for surface marker characterization

ExoView 200 platform (NanoView Biosciences) was employed to assess the expression of CD81 and CD9 [10]. Briefly, capture antibodies recognizing CD81 and CD9 were immobilized on chips to capture EVs from debris-free culture supernatants. Subsequently, the chips were finally incubated with detection antibodies, specifically AF555-anti-CD81 and AF488-anti-CD9, for quantitative analysis.

### EV labeling and in vitro internalization

To assess cellular uptake, EVs were labeled with DiD (1 µM, Solarbio) and reisolated. Recipient cells were incubated with these DiD-labeled EVs (200 µg/mL) for 4 h, followed by fixation with 4% paraformaldehyde (Elabscience). Cellular internalization was visualized with confocal microscope (LSM880, Zeiss) and quantified via flow cytometry (Millipore Guava easyCyte) [23, 24].

### EV labeling and in vivo tracing

To examine the biodistribution of DiR-labeled EVs, they were tagged with DiR (B8806, APExBIO) and administered intravenously. Four hours post-injection, key organs including the liver, spleen, blood, heart, and gastrocnemius muscle were harvested. DiR fluorescence was subsequently quantified using an in vivo spectrum imaging system (PerkinElmer) [25].

### Genome-wide RNAseq

DC2.4-derived EVs were administered intravenously into C57BL/6 mice for 24 h. Subsequently, hepatic CD11b^+^ Kupffer cells were isolated into VeZol Reagent (R411, Vazyme) using flow cytometry and subjected to RNA sequencing on DNBSEQ2000 (BGI Genomics, Shenzhen) [26, 27]. Transcript abundances were quantified using the Kallisto program (version 0.48.0) under default settings. Functional enrichment analysis within the Reactome Pathway was conducted using the “clusterProfiler” R package (version 4.2.2), and gene set variation analysis (GSVA) was performed with the “GSVA” R package. Adjusted *p-*value < 0.05 was set as the threshold for statistical significance.

### Real-time qPCR

Total RNA was extracted from DV-primed RAW264.7 cells with RaPure Total RNA Micro Kit (R4012, Magen) and reversely transcribed into cDNA using the HiScript IV RT SuperMix for qPCR (+ gDNA wiper) (R423-01, Vazyme). Real-time qPCR was performed using Hieff UNICON Universal Blue qPCR SYBR Green Master Mix (11184ES03, Yeasen) on StepOne Plus Realtime PCR system (Applied Biosystems). The values for specific genes were calculated as 2−(^ΔΔ^CT) and normalized to *18S*. Primer sequences are listed below: *Pde2a*: forward: 5’-ACGCGCAACATTCTCTGCTTCC-3’, reverse: 5’-TGCCACAGTAGATGGAGAAGGC-3’, *Pkd1*: forward: 5’-GATCAGACACCGCTCAACTTCC-3’, reverse: 5’-ACACCAGCTTCTAGGCGTTCCA-3’, *Cpeb2*: forward: 5’-GAGATCACTGCCAGCTTCCGAA-3’, reverse: 5’-CAATGAGTGCCTGGACTGAGCT-3’, *Malt1*: forward: 5’-GAACTGAGCGACTTCCTACAGG-3’, reverse: 5’-AACTGTCCAGCCAACACTGCCT-3’, *Creb5*: forward: 5’-GCAAGGTCCAAACCTCAGCAAC-3’, reverse: 5’-TGTCCGATGGTGCTCATGTTCC-3’, *18 S*: forward: 5’-GTAACCCGTTGAACCCCATT-3’, reverse: 5’-CCATCCAATCGGTAGTAGCG-3’.

### Myocardial infarction and phenotypic evaluation

All procedures involving animals were conducted under the Guidelines for the Care and Use of Research Animals as set by Soochow University. The Murine MI model was established on male C57BL/6 mice by ligating the left anterior descending artery [10, 25]. Cardiac function was continuously monitored using a Vevo 2100 imaging system equipped with a 30-MHz transducer (Visualsonics) [28, 29]. Transthoracic echocardiography was employed to acquire M-mode images, assessing left ventricular ejection fraction (EF), fractional shortening (FS), and thicknesses of the posterior wall in systole (LVPWs) and diastole (LVPWd). On Day 28 post-MI, ischemic hearts were harvested for Masson’s trichrome staining [30, 31].

### Hindlimb ischemia and phenotypic evaluation

The murine hindlimb ischemia model was established on male C57BL/6 mice [32]. Briefly, the femoral artery in the left hindlimb was carefully separated from the adjacent vein and nerve and then ligated. Following ischemia induction, specified EVs were administered, and hindlimb blood flow was monitored using a laser Doppler perfusion imager. The left gastrocnemius muscle was harvested on Day 21 post-ischemia for histological evaluation.

### “Engage & Evasion” strategy

In the implementation of the “Engage & Evasion” strategy in vivo, an initial dose of DV (50 µg/mouse) was administered, followed four hours later by a second dose of therapeutic MV^47^ (200 µg/mouse). A total of four injection sets were given for both MI and hindlimb treatment. In contrast, control groups were treated with equivalent doses of either MV^VEC^ or MV^47^. Similarly, for the in vitro approach, an initial DV dosage (50 µg/µL) was administered and subsequently followed, after four hours, by a therapeutic dose of MV^47^ (200 µg/µL).

### Immunohistochemistry and immunofluorescence

Immunohistochemical staining was conducted on paraffin-embedded sections of ischemic tissue [33, 34]. Briefly, 5-µm thick sections were subjected to deparaffinization, antigen retrieval, and permeabilization. They were then incubated with anti-mouse CD31 (ZA0063, ZuoChengBio) overnight, followed by HRP-conjugated secondary antibody. The slides were treated with DAB substrate to produce a brown coloration, after which nuclei were counterstained with hematoxylin for contrast. For immunofluorescence, HyperFluor 488 Goat Anti-Rabbit IgG (H + L) Antibody (K1206, APExBIO) was used as secondary antibody.

### Wound-healing assay

Wound-healing assay was performed to evaluate cell migration capability [35, 36]. Briefly, 2 × 10^5^ HUVECs were cultured in 6-well plates to form a confluent monolayer. Artificial wounds were generated using sterile pipette tips. Floating cells were subsequently removed with DPBS (6062011, Dakewe), and the adherent HUVECs were exposed to designated conditional medium. Photographic records were captured at 0, 6, and 12 h post-scratching. Wound area was quantified using Image J software and the percentage of closure was calculated to evaluate migration efficiency.

### Proliferative EdU assay

Cell proliferation was quantified with the EdU In Vitro Imaging Kit (KGA9609, Keygen BioTECH) [37]. Briefly, cells in logarithmic growth phase were seeded into 6-well plates at a density of 2 × 10^5^ cells/mL and subjected to designated treatments. Subsequently, pre-treated HUVECs were exposed to 10 µM EdU for 2 h to facilitate EdU integration. Subsequently, nuclear EdU was detected by binding fluorescence-labeled azide to its alkyne group. Finally, cells were stained with DAPI-containing anti-fade solution, and images were acquired via an inverted fluorescence microscope (Olympus). Quantification of EdU incorporation was performed using ImageJ, counting randomly across high-power fields.

### Tube formation assay

Ceturegel Matrix LDEV-Free Matrigel (40183ES08, YEASEN) was applied to coat 48-well plates with 4 × 10^4^ HUVECs per well, creating a matrix membrane. Digested HUVECs were then seeded into the plates and treated with the indicated conditional medium. Tube formation was monitored after 24 h of culture, with both the number and length of tubes quantified [38, 39].

### In vivo toxicity evaluation

To evaluate the hepatorenal toxicity of our “Engage & Evasion” strategy, serum was collected on Day 14 post-therapy. Serum concentrations of alanine aminotransferase (ALT; BC1555, Solarbio), aspartate aminotransferase (AST; BC1565, Solarbio), blood urea nitrogen (BUN; BC1535, Solarbio), and creatinine (Cr; C011-2-1, Nanjing Jiancheng) were quantified using specific kits [40]. Measurements were conducted using a multifunctional microplate reader (BIO-TEK). Additionally, to assess organ toxicity, Hematoxylin & Eosin (H&E) staining of brain, lung, liver, spleen, and kidney sections was performed to determine the extent of tissue damage (G1120, Solarbio) [41, 42].

### Statistical analyses

Data are reported as mean ± SEM. All statistical evaluations were performed using GraphPad Prism software, with a significance level established at *P* < 0.05. Comparisons between two groups utilized a two-tailed Student’s t-test, while comparisons involving multiple groups were analyzed using either one-way or two-way ANOVA.

## Electronic supplementary material

Below is the link to the electronic supplementary material.

Supplementary Material 1

## Data Availability

The raw sequencing data from this study have been deposited in the Genome Sequence Archive in BIG Data Center (https://bigd.big.ac.cn/), Beijing Institute of Genomics (BIG), Chinese Academy of Sciences, under the accession number: CRA016382.
