# Supplementary material for: An integrated “Engage & Evasion” approach for mononuclear phagocyte system escape and efficient extracellular vesicle therapy
Source: J Nanobiotechnology. 2024 Dec 19;22:770. doi: 10.1186/s12951-024-03032-z (PMC11658260; doi:10.1186/s12951-024-03032-z)
Supplement: Supplementary file 1 — Supplementary Material 1 [file 12951_2024_3032_MOESM1_ESM.pdf]

Supplementary data

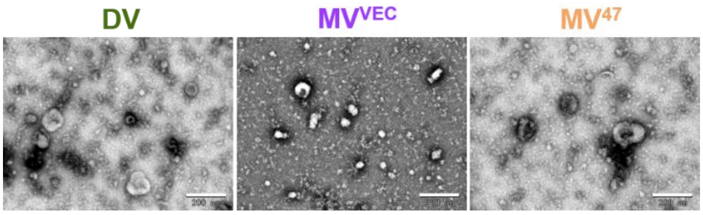

Figure S1. TEM image for EV morphology at lower scale. Scale bar = 200 nm.

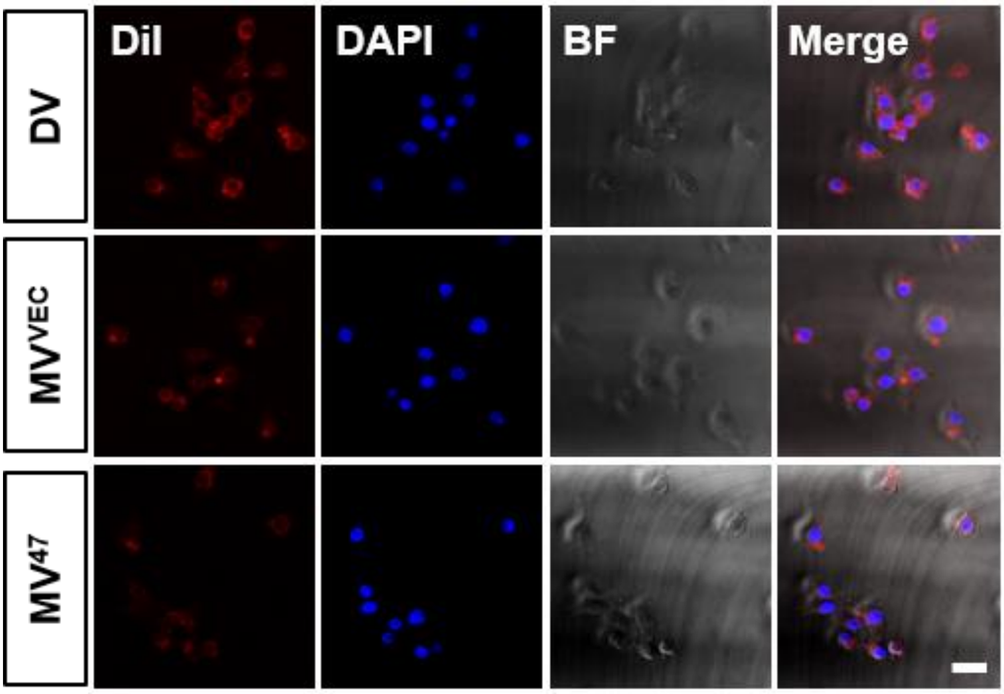

Figure S2. Differential Uptake of DV and MV^{47} by Macrophages. RAW264.7 macrophages were incubated with DiI-labeled EVs for 4 h. Representative images showing EV uptake. Scale bar = 20 µm.

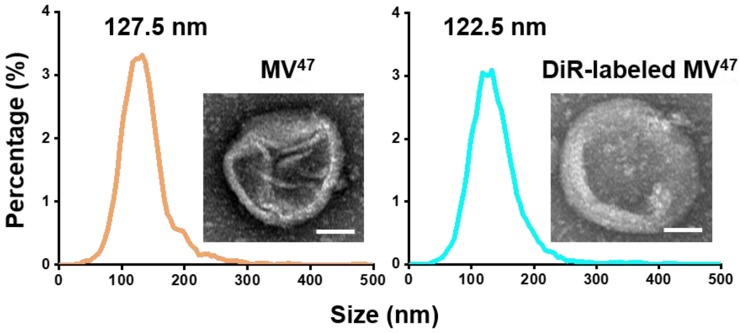

Figure S3. Characterization of DiR-labeled MV^{47}. Morphology and size distribution of DiR-labeled EVs were examined using transmission electron microscopy (TEM) and nanoparticle tracking analysis (NTA), respectively. Scale bar = 50 nm.

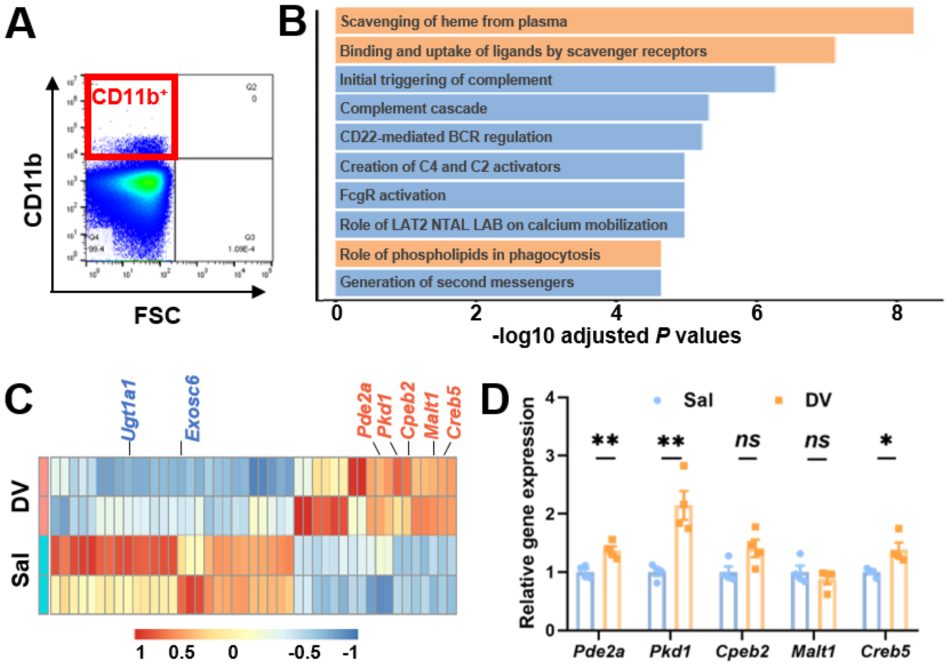

Figure S4. RNAseq analysis of DV-primed Kupffer cells. (A) Schematic illustration depicting the CD11b^{+} Kupffer cell sorting pattern. (B) Macrophage activity-related Reactome pathway analysis in DV-primed Kupffer cells, color-coded to indicate phagocytosis-related pathways (orange). (C) Hierarchical clustering of differentially expressed genes between control and DV-primed Kupffer cells 24 hours post-injection, with red and blue colors representing upregulated and downregulated genes, respectively. (D) mRNA expression of selected RNAseq candidates in DV-primed RAW264.7 cells (n = 4). Statistical evaluations were conducted using unpaired Student's t-test. Results were presented as mean ± SEM (* $P < 0.05$; ** $P < 0.01$; *ns*: not significant).

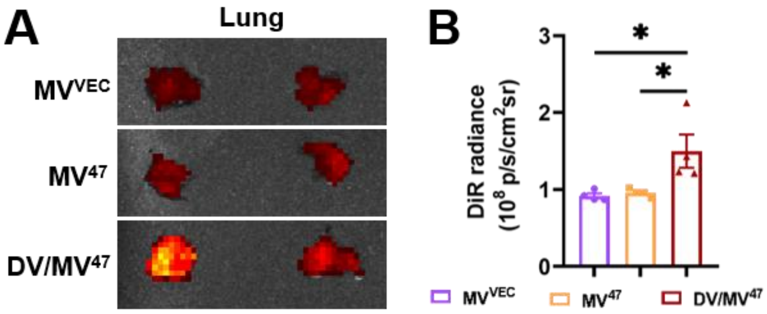

Figure S5. *In Vivo* Biodistribution of Therapeutic MV^{47}. Representative DiR optical images alongside quantification of biodistribution in the lung (*n* = 4). Statistical evaluations were conducted using one-way ANOVA. Results were presented as mean ± SEM (* $P < 0.05$).
